# Evaluation of a Bio-Based Solvent Pretreatment for Sustainable Froth Flotation of Black Mass from Spent Lithium-Ion Batteries

**DOI:** 10.1021/acssusresmgt.5c00058

**Published:** 2025-06-05

**Authors:** Aliza Marie Salces, Marc Simon Henderson, Alvaro José Rodríguez-Medina, Kai Bachmann, Elsayed Oraby, Chau Chun Beh, Martin Rudolph, Jacques Eksteen, Anna Vanderbruggen

**Affiliations:** † Helmholtz Zentrum Dresden Rossendorf (HZDR), 557386 Helmholtz Institute Freiberg for Resource Technology (HIF), Chemnitzer Straße 40, 09599 Freiberg, Germany; ‡ 137665 Université de Lorraine, GeoRessources, 54000 Nancy, France; § Western Australian School of Mines: Minerals, Energy and Chemical Engineering, 1649 Curtin University, Perth, Western Australia 6102, Australia; ∥ Future Battery Industries CRC, Bentley, Western Australia 6102, Australia; # ERZLABOR Advanced Solutions GmbH, Chemnitzer Straße 40, 09599 Freiberg, Germany

**Keywords:** flotation, black mass, PVDF binder, graphite, dihydrolevoglucosenone, Cyrene, pyrolysis, characterization

## Abstract

Froth flotation effectively separates anode graphite from cathode active materials (CAMs) of spent lithium-ion batteries when CAM particles are free of organic binders, such as polyvinylidene fluoride (PVDF). This study investigated a bio-based solvent, dihydrolevoglucosenone (Cyrene^TM^ ), as a pretreatment to remove the PVDF binder from both single chemistry black mass (BM) and industrially produced mixed chemistry black mass (IBM). The subsequent flotation combined with high-intensity attritioning improved CAMs and graphite separation efficiency compared to that of mechanical pretreatment alone, increasing from 0.30 to 0.53 in BM and from 0.37 to 0.54 in IBM. Although pyrolysis resulted in higher separation efficiencies of 0.92 in BM and 0.78 in IBM, Cyrene pretreatment presents advantages in non-emission of toxic gases and in preserving lithium within the CAMs. In the flotation process water, an average lithium dissolution of only 5.5% in BM and 14.7% in IBM was recorded with Cyrene pretreatment, compared to that of 29.3% in BM and 55.4% in IBM with pyrolysis pretreatment. The lower quality of the flotation products obtained with Cyrene pretreatment necessitates further purification steps such as cleaner flotation. Optimizing pretreatment parameters is crucial, including the Cyrene to black mass ratio and contact time. A key challenge is preventing the thermally induced phase separation of PVDF at temperatures lower than 80 °C, which negatively affects the effective separation of graphite and CAMs by froth flotation.

## Graphical abstract

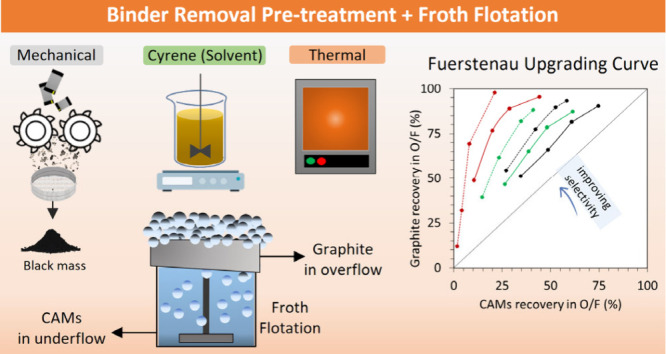

## 1 Introduction

There has been a growing interest in applying traditional mineral processing techniques, such as froth flotation, for the separation of black mass components of lithium-ion batteries (LIBs) before downstream metallurgical recycling processes. Black mass is a fine powder containing cathode active materials (CAMs) and anode active materials (i.e., graphite) obtained after mechanical processing (i.e., shredding, sieving, and/or sifting). The application of froth flotation is feasible as CAMs and graphite particles are reported to have significant differences in their wettabilities: hydrophilic and hydrophobic, respectively.^1,2^ However, the presence of polymeric binders such as polyvinylidene fluoride (PVDF), which glues the active material together for a proper layer formation onto the electrodes, leads to loss of this wettability contrast (i.e., CAMs become hydrohopic). For efficient separation by froth flotation, it becomes crucial to remove the PVDF binder.

Several researchers have proposed various techniques for binder removal before froth flotation, including mechanical and thermal pretreatments as summarized in the work of Hong et al.^3^ Thermal pretreatments at 400 °C to 600 °C in a vacuum, inert gas, or air have demonstrated effectiveness in removing the PVDF binder and in improving flotation efficiency.^4^ Emerging thermal pretreatment techniques using plasma^5,6^ and microwave^7^ have also been reported.

The persistent concern surrounding PVDF removal by thermal treatment is the generation of toxic off-gases such as hydrogen fluoride, polynuclear aromatic hydrocarbons, and halogenated hydrocarbons.^8^ Apart from this, pyrolysis of black mass results in carbothermic reduction of CAMs,^9^ which hinders the possibility of direct recycling. The CAMs in pyrolyzed LIBs or black mass are more susceptible to lithium loss when the subsequent recycling processes are water-assisted.^10–14^

The chemical approach to solubilize and remove the PVDF binder could be an alternative. The use of Fenton’s reagent (Fe^2+^/H_2_O_2_) was proposed to oxidize the PVDF coating prior to froth flotation.^15–17^ However, a pH of 2.5–3.5 is needed for Fenton’s reagent to be effective, requiring acid for pH modification. Other known solvents for PVDF fall under the dipolar aprotic category (e.g., *N*-methyl-2-pyrrolidone and *N*,*N*-dimethylformamide);^18^ however, their use is limited due to hazards to human health and the environment . However, research investigations on the dissolution of PVDF using green solvents are ongoing as summarized by Marshall et al.^18^ Most of these identified solvents have also been investigated for the removal of PVDF from battery materials, particularly toward the delamination of CAMs from the aluminum (Al) foil. Fu et al.^19^ investigated the use of supercritical CO_2_ (ScCO_2_) combined with the co-solvent dimethyl sulfoxide (DMSO) at optimum conditions of 70 °C and 80 bar for the delamination of lithium managese oxide. The dissolved PVDF reprecipitated from the co-solvent at room temperature and pressure. Similary, Hayagan et al.^20^ explored the use of ScCO_2_ and acetone as co-solvents, in conjunction with triethyl phosphate (TEP) at operating conditions of 120 °C and 100 bar for the delamination of lithium cobalt nickel manganese oxide (NMC). Post-dissolution, the acetone-TEP-PVDF solution was separated through filtration, and PVDF was recovered by re-precipitation from the solution through the addition of water. Wang et al.^21^ utilized fatty acid methyl esters (FAME) at 190 °C to delaminate lithium iron phosphate. However, PVDF was not recovered from the FAME solvent. Bai et al.^22^ proposed the use of dihydrolevoglucosenone (Cyrene^TM^) for PVDF dissolution at 100 °C to delaminate NMC cathode scrap. The PVDF-Cyrene solution was separated from CAMS by hot filtration, and PVDF was recovered via thermally induced separation. Similarly, Elmaataouy et al.^23^ reported the use of glycerol-triacetate for delamination of lithium nickel cobalt aluminum oxides.

These previous studies have primarily focused on the use of a solvent for PVDF removal to delaminate CAMs from the Al foil. However, to the best of the authors’ knowledge, green solvents have not been reported as a potential pretreatment for froth flotation black mass beneficiation. Therefore, this work investigated the use of Cyrene for the solubilization of PVDF directly from electrode active particles, specifically CAMs. From a sustainability perspective, Cyrene is an ideal candidate, as it is derived from plant-based biomass. Its key properties are detailed in these studies.^24,25^ Unlike conventional dipolar aprotic solvents, Cyrene exhibits no significant toxicity, is non-mutagenic, non-genotoxic, and readily biodegradable within 28 days.^26^

This study employed single chemistry black mass (BM) and industrially produced mixed chemistry black mass (IBM) to compare the flotation separation efficiency of graphite and CAMs pretreated with Cyrene to that of subjected to purely mechanical and thermal pretreatments. The BM served as a reference due to its simple and known composition, and the IBM provided the complexity a typical recycler can face due to its industrial origins with an unknown binder, mix of cathode and anodes, and higher content of Al, Cu, and casing impurities. To understand PVDF binder removal, characterization techniques, such as time-of-flight secondary ion mass spectrometry (ToF-SIMS), scanning electron microscopy (SEM), and thermogravimetric analysis (TGA), were implemented. The ultimate goal is to establish the groundwork for a benign flotation pretreatment of black mass before subsequent recycling processes and further toward direct recycling of both graphite and the CAMs.

## 2 Materials and Methods

### 2.1 Materials and Pretreatments

For this study, three sets of materials were examined, as listed in Table S.1 (in the Supporting Information). To create a model black mass (Model-BM) simulating binder-free and completely liberated particles, pristine NMC111 (MSE supplies, Product No. PO0126) and spheroidized natural graphite (ProGraphite GmbH, product No. 1112-1) powders were mixed at an 80:20 mass ratio. A single chemistry black mass (BM) was prepared in a similar ratio to that of model-BM. Both the cathode (NMC111 with PVDF + Al foil) and anode (spheroidized natural graphite with water-soluble SBR-CMC + Cu foil) material were obtained from manually dismantled prismatic hard case LIB cells, described in Werner *et al*.^27^ To obtain the anode and cathode powders, the anodic and cathodic components were crushed separately using a Turbo-Rotor mill followed by sieving at 0.5 mm. Additionally, mixed chemistry industrial black mass (IBM) was provided by Envirostream Australia Pty Ltd. The material is sourced from consumer batteries with dominant chemistries of lithium cobalt oxide (35.8%) and lithium nickel manganese cobalt oxide (62.0%), along with lithium iron phosphate (2.2%), as shown with the particle-based analysis (MLA) in Figure 1.

**Figure 1:**
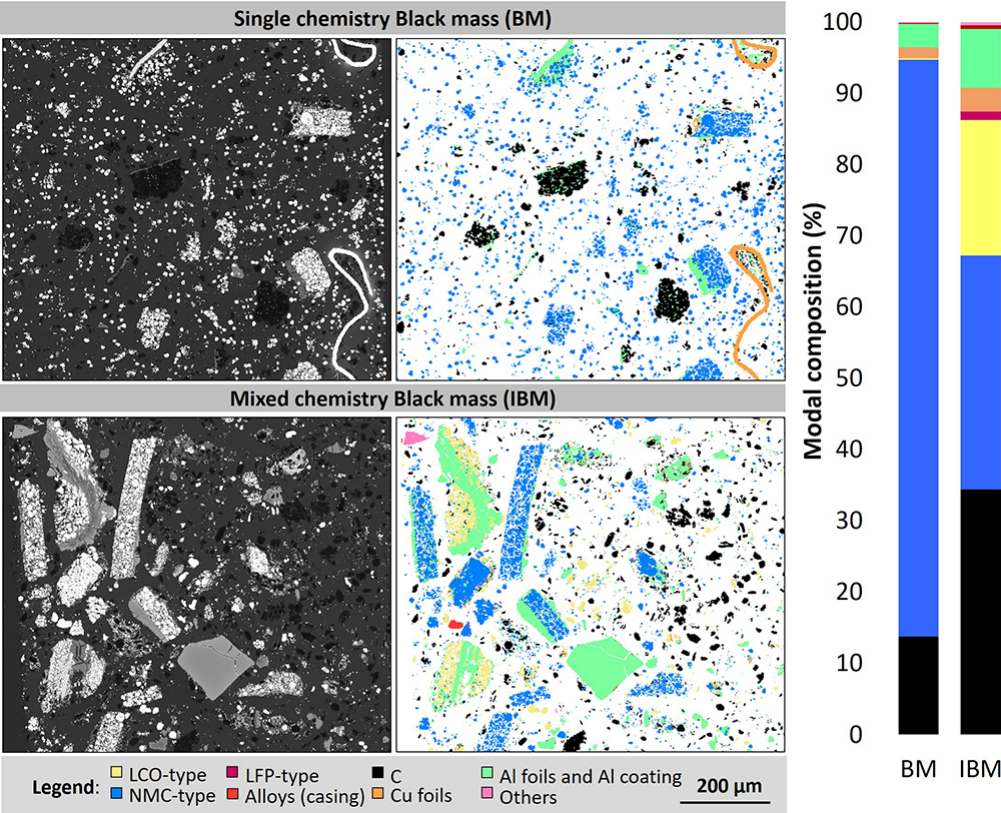
(a) BSE images and processed images by MLA for the BM and IBM and (b) modal composition of the BM and IBM obtained by MLA using battery component grouping.

The BM contains 18.7% C, 5.6% Li, 15.7% Co, 15.1% Mn, 15.3% Ni, 0.9% Al, and 1.6% Cu, and the IBM contains 39.3% C, 2.8% Li, 12.9% Co, 3.3% Mn, 10.4% Ni, 3.4% Al, 2.1% Cu, and 0.1% Fe. The complete elemental composition (Table S.2), particle size distribution (Table S.3 and Figure S.1), and SEM micrographs (Figures S.2-S.4) are available in the Supporting Information.

In their original condition, the black masses were ‘mechanically treated’ (M-BM and M-IBM) and subjected only to mechanical shredding and sieving. The Cyrene pretreatment was achieved following the work of Bai et al.^22^ About 80 g of M-BM or M-IBM was mixed with 400 mL of Cyrene (99.3% dihydrolevoglucosenone, batch no. DCyD12_220308, Circa-FC5) at 25 °C. An overhead stirrer was used to intensively mix the black mass and Cyrene while heating at 100 °C for 1 h. This was followed by vacuum filtration at elevated temperature (90 °C–95 °C) inside a drying oven to prevent the re-precipitation of PVDF when the solution cools below 80 °C. To remove residual Cyrene, the recovered solids were subsequently washed with hot deionized water (90 °C–95 °C). The resulting black mass is now termed MC-BM and MC-IBM, respectively. M-BM and M-IBM were also pyrolyzed at 550 °C under nitrogen atmosphere for 3 h to produce MT-BM and MT-IBM, respectively. Figure 2 summarizes these pretreatments.

**Figure 2:**
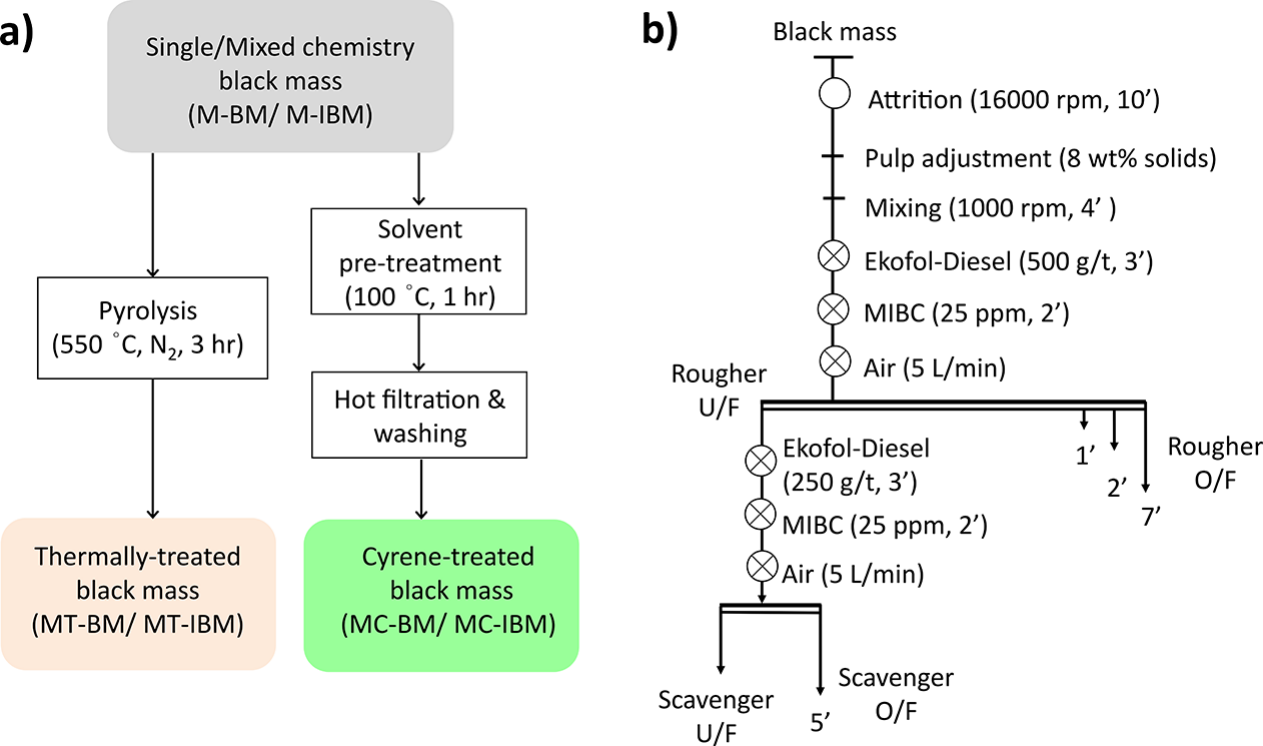
Schema of the different pretreatments for (a) binder removal and (b) the flotation process.

### 2.2 Characterization

#### 2.2.1 Bulk and Particle-Based Analysis

The elemental compositions of the BM and IBM, and their flotation products, were quantified by inductively coupled plasma-atomic emission spectroscopy (ICP-OES, Optima 8300, PerkinElmer). About 0.2 g sample was digested with aqua regia (2 mL of HNO_3_ and 6 mL of HCl) for the analysis. The carbon content was determined by total combustion using PerkinElmer Series II CHNS/O Analyzer 2400. The carbon content represents graphite, PVDF/residues, and a potentially polymeric separator depending on the pretreatment of the black mass. The lithium concentration in the flotation process water was measured using flame-atomic absorption spectroscopy (F-AAS, contraAA 700, Analytik Jena).

The particle size distribution was determined by using a laser diffractometer (HELOS, Sympatec GmbH, Germany). The phase composition and geometrical parameters (e.g., size, shape, and association) of particles in the BM and IBM were evaluated by using mineral liberation analysis (MLA), a SEM-based automated image analysis system. This system comprises a SEM (FEI Quanta 650F) equipped with two EDX spectrometers (Bruker Quantax X-Flash 5030), and data acquisition was automated using MLA Suite 3.1.4 software. For this analysis, the black mass samples were prepared in an iodine epoxy resin following the methodology of Vanderbruggen *et al*.^28^ Additionally, SEM images of the black masses were acquired for qualitative evaluation by fixing loose powder samples onto a carbon patch.

#### 2.2.2 PVDF and Its Residues after Pretreatments

To determine the extent of PVDF binder removal after the Cyrene pretreatment, ToF-SIMS and TGA were conducted. TOF-SIMs (M6 instrument, IONTOF GmbH, Münster, Germany) was performed to determine the presence of PVDF or its residue on the black mass particles’ surfaces. A detailed procedure on sample preparation and methodology including characteristic peak identification of PVDF have recently been reported in Henderson et al.^29^ Thermogravimetric analysis (TG 309 Classic, NETZSCH, Germany) was conducted to quantitatively determine the residual organic content of the pretreated black masses. To do this, about 40 mg sample was heated to 650 °C in a nitrogen atmosphere at a heating rate of 20 K/min.

### 2.3 Flotation Experiment

Flotation was conducted using a laboratory-scale mechanically stirred flotation device (GTK Labcell from Outotec) in a 1-L cell with an automated scraping system. Given the limited quantities of black mass samples and Cyrene, the flotation experiment was conducted without replicates. However, minimal variance is expected due to careful control in flotation parameters, as demonstrated in four previous black mass flotation tests.^30^ The parameters were fixed also based on this previous work. For attrition, a high shear dispersing instrument (T25, IKA ULTRA-TURRAX®) was utilized at an agitation speed of 16,000 rpm for 10 min. After each pretreatment, 80 g sample was dispersed in tap water. An emulsion of Ekofol 440 (Floc-Tech GmbH, Germany) and diesel was added as a graphite promoter at a dosage of 500 g/t in the rougher stage and 250 g/t in the scavenger stage, aimed at increasing the hydrophobicity of graphite. The emulsion was prepared by adding the required amount of the promoter to 100 mL of water and intensively mixing at 10,000 rpm for 30 s using the same dispersing instrument. Methyl isobutyl carbinol (MIBC, 99% C_6_H_14_O, Alfa Aesar, Product No. A13435) was used as a frother at a concentration of 25 ppm. The airflow rate and impeller speed were kept constant at 5 L/min and 1000 rpm, respectively. The overflow products (O/F) were collected after 1, 2, and 7 min during the rougher stage and 5 min for the scavenger stage. After each flotation experiment, the mass of collected froth was measured wet and after drying in an oven (45 °C for 12 h) to determine mass and water recovery. Finally, representative samples were analyzed for their carbon and metal content. The underflow product (U/F) was processed accordingly. The flotation recoveries and grades were calculated using eq 1 and eq 2

1 $$R_{graphite}=\frac{\overset{n}{\underset{i=1}{\sum }}\left(C_{i}\cdot c_{i,graphite}\right)}{F\cdot f_{graphite}}$$

2 $$G_{graphite}=\frac{\overset{n}{\underset{i=1}{\sum }}\left(C_{i}\cdot c_{i,graphite}\right)}{\overset{n}{\underset{i=1}{\sum }}C_{i}}$$

where *R* and *G* are the recovery and grade of the component of interest (i.e., graphite and CAMs)*, respectively*, *C*_*i*_ is the O/F mass, *c*_*i*_ is the grade (concentration by mass) of the component of interest in the O/F, *F* is the feed mass, and *f* is the grade of the component of interest in the feed. The grade-recovery curve and Fuerstenau upgrading curve^31^ were used to visualize the flotation separation efficiency. The cumulative separation efficiency (CSE) was calculated using eq 3, modified from Wills and Finch^32^

3 $$CSE=\overset{n}{\underset{i=1}{\sum }}\frac{R_{i,graphite}-R_{i,CAMs}}{R_{i,graphite}}$$

where R_i,graphite_ and *R*_*i,CAMS*_ are the recoveries of the graphite and CAMs in the O/F. A CSE of 1 is the ideal separation of graphite and CAMs.

## 3 Results and Discussion

### 3.1 Ideal Flotation Behavior of Liberated and Binder-Free Particles

The flotation of pristine graphite and NMC was investigated to determine the effect of Cyrene pretreatment (i.e., heating at 100 °C and solvent pH ∼ 1.8) on their pristine flotability. After individual flotation of the Cyrene-treated graphite and NMC, the obtained process water has pH values of 7.7 and 8.0, respectively. As much as 99.6% of the graphite was recovered in the O/F, while only 4.2% NMC was recovered in the O/F. Meanwhile, flotation of Cyrene-treated model-BM resulted in a recovery of 93.7% graphite in the O/F and even 0.4% NMC in the O/F. The graphite concentrate achieved a grade of 97.2% C.

### 3.2 Single Chemistry Black Mass

Figures 3a and 3b present the C (assumed henceforth as graphite) and NMC (calculated as Ni + Co + Mn) recoveries over time in the rougher-scavenger stage, respectively. In MC-BM, the flotation of graphite is rapid, with about 37.8%–43.3% recovered in 3 min and about 70.1%–81.4% recovered after 10 min. For NMC, flotation is gradual, with 32.6%–38.9% recovery after 10 min. Despite increasing the graphite recovery to about 86.1%–91.1%, the subsequent scavenging stage with an additional dosage of flotation reagents further resulted in an increased NMC recovery in the O/F to 42.6%–56.5%. Evidently, there is a clear distinction between the recovery curves of NMC, with MC-BM situated between M-BM and MT-BM. The attrition pretreatment only resulted in a decrease in NMC recoveries in MC-BM and MT-BM.

**Figure 3:**
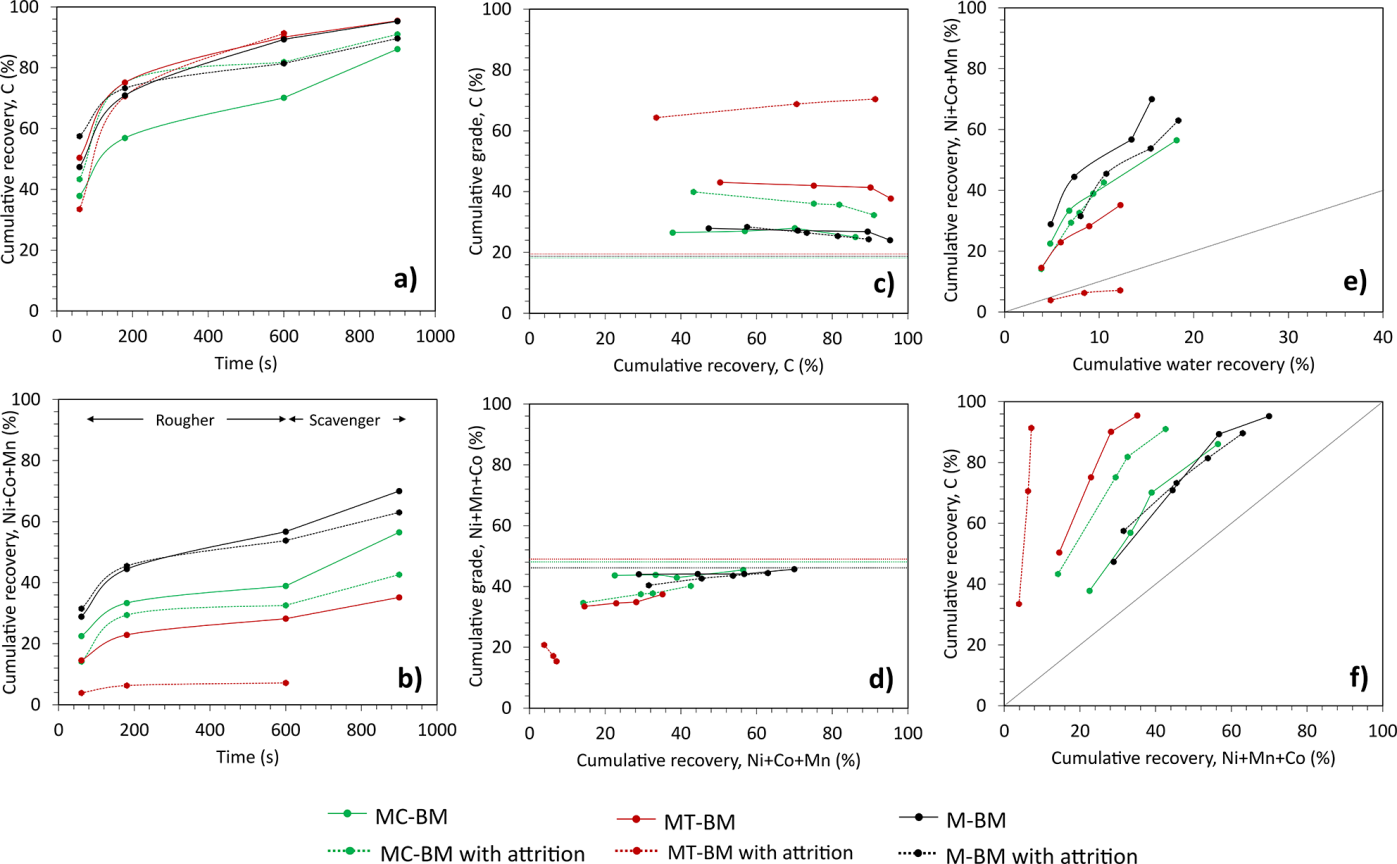
Recovery over time in the overflow product of (a) Graphite and (b) NMC. Grade-recovery curve in the overflow product of (c) Graphite and (d) NMC. Horizontal lines correspond to initial carbon and NMC grade in the feed. (e) NMC recovery vs water recovery. (f) Fuerstenau upgrading curve.

Figures 3c and 3d show the grade-recovery curves of graphite and NMC in the O/F. As mentioned, high graphite recoveries were achieved in M-BM, MC-BM, and MT-IBM. The impact of the binder removal strategies is more evident in the reduction of NMC recovery in the O/F. NMC grade and recovery in MC-BM with attritioning are 40.1% and 42.6%, respectively. This represents an improvement over the M-BM with attrition, which has an NMC grade and recovery of 44.4% and 63.0%, respectively. The high recoveries of NMC in the O/F resulted in minimal improvement in graphite grade with only 32.3% C in MC-BM with attritioning and 24.3% C in M-IBM with attritioning from an initial 18.7% C in the black mass feed. As a benchmark, MT-BM with attritioning obtained a graphite grade of 70.4% C.

Following PVDF removal, NMC is assumed to become predominantly hydrophilic; thus, its recovery in the O/F is primarily attributed to water entrainment.^1^ In Figure 3e, all the black masses, except for MT-BM with attritioning, are situated above the bistrate line, indicating the prevalence of true flotation through attachment to air bubbles via collector/organic binder interaction on the surfaces of NMC particles. The Fuerstenau upgrading curve in Figure 3f shows that flotation selectivity is slightly improved with Cyrene pretreatment with attritioning compared to mechanical pretreatment alone.

### 3.3 Mixed Chemistry Black Mass

Figures 4a and 4b show the graphite and mixed CAMs (reported as Ni+Co+Mn containing particles) recovery over time, respectively. In MC-IBM, the flotation of graphite is rapid, with about 39.2%–46.6% recovered in 3 min and about 61.2%–66.0% recovered after 10 min. All pretreated IBM exhibited similar graphite recovery curves, except for MT-IBM with attritioning, which shows remarkably slow graphite flotation. The slow graphite flotation with attritioning from thermal-treated black mass is a recurring phenomenon, as cited in previous study.^10^

**Figure 4:**
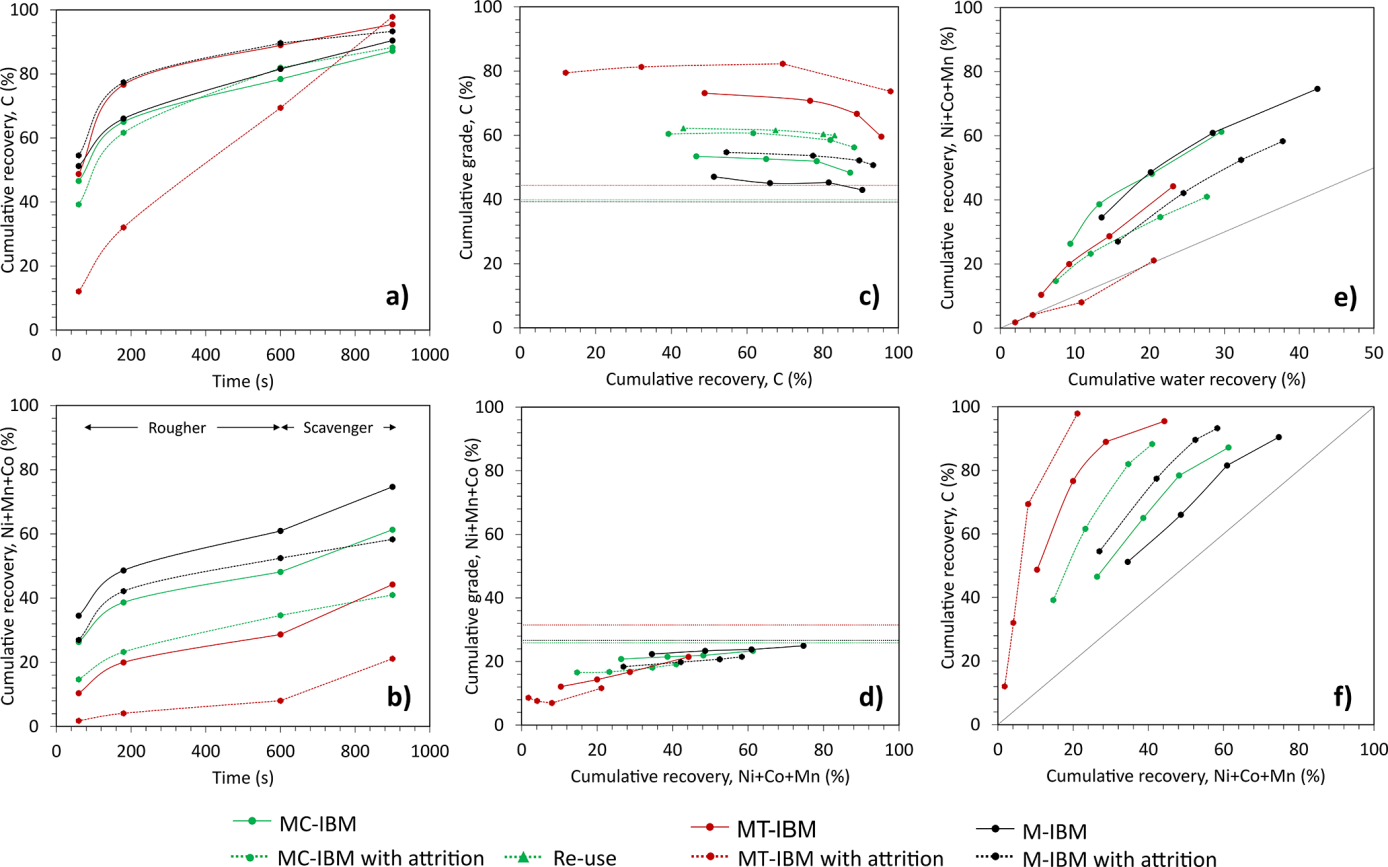
Recovery over time in the overflow products of (a) Graphite and (b) CAMs. Grade-recovery curve in the overflow product of (c) Graphite and (D) CAMs. (e) CAMs recovery vs water recovery. (f) Fuerstenau upgrading curve.

In MC-IBM, the CAMs flotation is slow to moderate, with 34.7%–48.2% recovery after 10 min. Furthermore, the addition of the scavenger stage further increased the recovery rate of graphite to about 87.2%–88.3%; however, this also increased the CAMs recovery in the O/F to 41.0%–61.3%. Similar to BM, there is also a clear distinction between the CAM recovery curves, with MC-IBM situated between M-IBM and MT-IBM. The attritioning pretreatment also resulted in a significant reduction in CAM recoveries in the O/F.

Figures 4c and 4d show the grade-recovery curves of graphite and CAMs. Similar to BM, the final graphite recoveries were comparable across different pretreatments. The effect of Cyrene pretreatment was more pronounced on the CAM recovery in the O/F. Specifically, MC-IBM with attritioning demonstrated a decrease in CAM recovery to 41.0% from 58.3% in M-IBM. This reduction of CAMS recovery in the O/F resulted in a graphite concentrate with a grade of 56.2% C in MC-IBM with attritioning and 50.7% C in M-IBM with attritioning, up from an initial 39.6% C. Investigation into Cyrene re-use indicates that the graphite grade achieved after flotation was comparable to that of fresh Cyrene, although a slight decrease in graphite recovery was noted.

The water recovery in Figure 4e revealed the potential role of true flotation on CAM recovery, with all black mass types except MT-IBM with attritioning situated above the bisector line. The SEM images (Figure S.5) of the rougher’s first concentrate show coarser CAM particles reporting to the O/F for M-IBM and MC-IBM; however, finer CAM particles were observed in MT-IBM, likely contributed by water entrainment. Finally, the Fuerstenau upgrading curve in Figure 4f highlights that Cyrene pretreatment resulted in an improvement in graphite-CAMs separation.

### 3.4 Residual Organics in the Pretreated Black Mass

In an attempt to determine the presence and removal efficiency of PVDF with different removal techniques, a ToF-SIMS analysis was employed. Prior research^29^ identified three characteristic peaks of PVDF, such as C_3_H_2_F_3_^+^ (95 m/z), C_3_HF_4_^+^ (113 m/z), and C_3_H_2_F_5_^+^ (133 m/z), which serve as an indicator of PVDF presence. Figure 5a-f shows these characteristic peaks in MC-BM and MC-IBM, indicating incomplete PVDF removal in these samples. Conversely, the absence of these peaks in MT-BM and MT-IBM implies effective PVDF removal; however, carbonaceous decomposition compounds of PVDF were expected to form on the surfaces of black mass as reported in previous work.^29^

**Figure 5:**
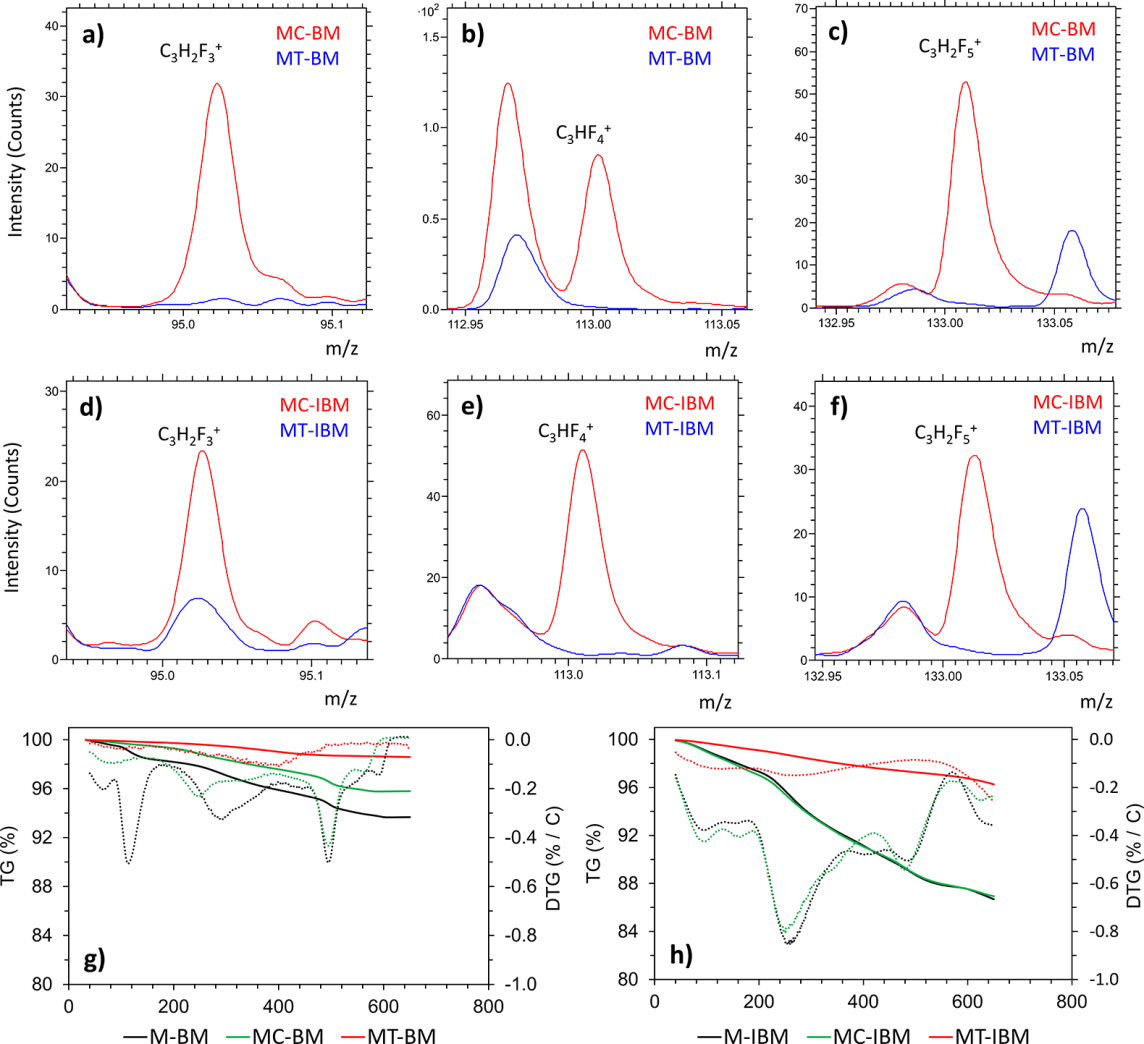
Comparison of the characteristic PVDF peaks in (a-c) MC-BM and MT-BM and (d-f) MC-IBM and MT-IBM. TG curves of the pretreated (g) BM and (h) IBM.

To quantify the residual organics including PVDF, TGA was performed. Figures 5g and 5h show mass losses of 4.2% and 12.3% in MC-BM and MC-IBM, respectively, while M-BM and M-IBM exhibited mass losses of 6.3% and 13.1%, respectively. Derivative thermogravimetry (DTG) curves of the BM revealed distinct mass loss events: 220 °C–250 °C in MC-BM (decomposition of residual Cyrene), 250 °C–350 °C in M-BM (decomposition of the anode binder SBR-CMC), and 470 °C–500 °C in both M-BM and MC-BM (decomposition of PVDF). The DTG curve of the MC-IBM showed a significant mass loss between 200 °C and 300 °C, likely attributable to residual Cyrene. However, in M-IBM, the similar mass loss event at this temperature range could point to an undetermined anode binder being responsible. Furthermore, a minimal decomposition peak was observed at 470 °C–500 °C in M-IBM and MC-IBM, potentially obscured by the preceding larger peak, suggesting the presence of residual PVDF.

### 3.5 Lithium in Process Water

Table 1 highlights the pH and lithium dissolution and concentration in the flotation process water after different binder removal pretreatments. In MT-BM, approximately 26%–32% of lithium was dissolved in the process water, resulting in concentrations ranging from 1100–1,300 mg/L. In MT-IBM, this dissolution increased substantially, with 55%–56% of lithium entering the water phase at concentrations of 1,400 mg/L–1,500 mg/L. M-BM and MC-BM exhibited lower lithium dissolution, ranging from 4.5% to 6.6%, while M-IBM and MC-IBM showed a dissolution range from 11.6% to 16.1%.

**Table 1:** Lithium Concentration and Dissolution in the Process Water after Different Binder Removal Pretreatments^a^

	pH of flotation water	Li in process water (mg/L)	Li dissolution (%)
M-BM	9.3	200–250	5.8–6.1
MC-BM	8.5	160–200	4.5–6.6
MT-BM	11.5	1,100–1,300	26.4–32.3
M-IBM	9.7	340–360	11.6–13.1
MC-IBM	8.5	280–340	13.4–16.1
MT-IBM	11.7	1,400–1,500	55.2–55.7

a The Li content was obtained by F-AAS.

### 3.6 Discussion

This study evaluated Cyrene pretreatment as a method for PVDF removal prior to froth flotation. The flotation of Cyrene-treated model-BM containing pristine NMC and graphite showed that the ideal flotation behavior of these particles was not affected by the Cyrene pretreatment with 93.7% graphite recovery and only 0.4% NMC recovery in the O/F and a resulting graphite concentrate purity of 97.2% C. The flotation of Cyrene-treated BM and IBM also resulted in high graphite recoveries, up to 90% in one rougher-scavenger stage. However, significant recoveries of CAMs indicate incomplete PVDF removal. Nevertheless, compared to mechanical pretreatment alone, Cyrene pretreatment demonstrated improved separation efficiency of graphite from CAMs, as evidenced by selectivity index analysis (Table 2). Tabulated grades and recoveries of graphite and CAMS are available in Tables S.4 and S.5 in the Supporting Information. The lower separation efficiency in the scavenger stage is contributed by a disproportionately larger increase in CAM recoveries compared to graphite recoveries. CAMs float more during the scavenger stage due to lower competition for the air bubbles, as the majority of graphite floats faster and has been recovered during the rougher stage. However, coarser graphite particles were also noted to be recovered during the scavenger. Thus, a scavenger stage is necessary to maximize graphite recovery in the O/F and reduce the number of graphite impurities from the CAMs in the U/F.

**Table 2:**
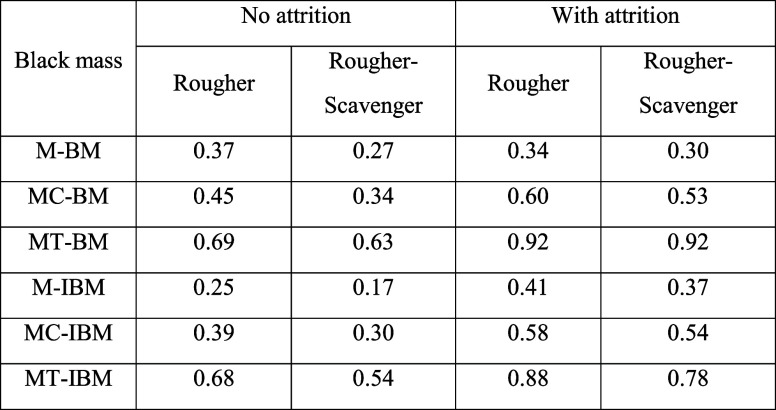
Cumulative Separation Efficiency^a^

a Low, red; Average, yellow; High, green.

While Cyrene has demonstrated effective PVDF solubilization in the presence of battery powders,^22,29,33^ the separation of the Cyrene-PVDF solution from the treated black mass is crucial. This separation necessitates hot filtration above 80 °C to prevent thermally induced phase separation of PVDF. The high viscosity of the Cyrene-PVDF solution, reaching 810 mPa·s as reported by Marshall et al.,^18^ prolongs filtration times, a problem also contributed to by the use of fine filter paper (<5 μm) to retain the black mass particles. This separation challenge is anticipated to scale with process volume, suggesting the potential need for alternative dewatering equipment or centrifugation.

With ineffective dewatering, re-deposition of PVDF onto particle surfaces occurs upon cooling of the Cyrene-PVDF solution. ToF-SIMS analysis confirmed the presence of PVDF on the surfaces of both MC-BM and MC-IBM. Furthermore, TGA revealed similar mass loss profiles for MC-IBM and M-IBM. Despite this, the enhanced flotation selectivity observed for MC-IBM compared to M-IBM implies an incomplete coating of PVDF on CAM surfaces. This re-deposited PVDF may result to aggregation of fine CAM particles, as previously observed,^29^ which could lead to reduced CAM recovery in the O/F by entrainment.

Thermal pretreatment (i.e., pyrolysis) has been established as a highly effective pretreatment for black mass flotation, consistently achieving CAM recoveries in the O/F ranging from 5% to 25% and graphite recoveries between 90% and 98% across numerous studies.^10,19,34–41^ Here, thermal pretreatment achieved minimum NMC and CAM recoveries of 7.1% and 21.1% in the O/F , respectively. However, a key advantage of Cyrene is that there is no emission of toxic fluoride-containing gases. Recovery of PVDF from Cyrene is feasible during cooling;^22,29^ however, the quality of the recovered PVDF remains to be thoroughly characterized. Furthermore, Cyrene demonstrates reusability for black mass pretreatment without compromising flotation separation efficiency under the tested conditions. Notably, Cyrene pretreatment preserves lithium within the CAMs, contrasting with pyrolysis, where considerable amounts of lithium (up to 55%) can leach into the process water reaching concentrations up to approximately 3 g/L.^42^ This concentration is comparable to lithium levels found in many global brines.^43^ Pyrolysis significantly alters the CAM phases,^4^ precluding direct recycling; thus, a further metallurgical process is required. Conversely, these findings suggest that Cyrene pretreatment of black mass offers potential for direct CAM recycling due to the absence of significant chemical transformations observed in pyrolysis-based pretreatments.

Direct utilization of recycled CAMs and graphite necessitates minimal impurity levels to not affect battery performance.^44^ Commercial battery-grade CAMs typically require impurity concentrations in the parts-per-million (ppm) range, while battery-grade graphite requires a purity of 99% C, comparable to the pristine materials used in this study. However, the graphite concentrate obtained from MC-BM contained only 32.3% C, with significant impurities including 40.1% NMC, 4.3% Li, 0.2% Al, and <0.1% Cu. Similarly, the CAM product in the U/F contained 59.8% NMC and 6.5% Li, with 3.5% C, 1.3% Al, and 3.2% Cu as impurities. In MC-IBM, the graphite concentrate has 56.2% C, with 19.1% NMC, 1.58% Li, 1.9% Al, and 1.4% Cu as impurities. The CAM product in the U/F contained 42.0% NMC and 3.7% lithium, with 11.4% C, 5.6% Al, and 2.9% Cu. These results highlight the need for further, potentially multi-stage, cleaning flotation and additional chemical purification^45^ and relithiation^22^ to meet specifications for direct battery material reintegration.

The heterogeneity and quality of the black mass can significantly influence the processing approach. Cyrene pretreatment is suited for black mass with homogeneous CAM chemistry or even battery cell scraps due to their higher purity, thus increasing the feasibility of direct recycling. The recovery of CAMs from complex and heterogeneous black mass is feasible; however, separation of different CAMS from each other presents a significant challenge for direct recycling. Such a material is preferentially suited for hydrometallurgical processing.

Overall, this study represents the initial application of Cyrene for PVDF binder removal prior to froth flotation. Operating parameters, including treatment temperature and time, the Cyrene-to-black mass ratio, and postfiltration steps, have not been optimized. Therefore, future research should focus on systematically optimizing these parameters to further improve the efficiency of the Cyrene pretreatment process.

## 4 Conclusion

In this work, the sustainable bio-based solvent dihydrolevoglucosenone (Cyrene^TM^) is proposed as an environmentally benign pretreatment step to remove PVDF from CAMs of spent LIBs and enable the effective froth flotation separation of graphite and CAMs. Results of froth flotation demonstrate that Cyrene pretreatment, combined with high-intensity attritioning, exhibits potential for achieving improved flotation selectivity compared to conventional mechanical pretreatment methods. While pyrolysis pretreatment yielded the most favorable flotation results, Cyrene offers a distinct advantage by eliminating the release of toxic off-gases. Furthermore, Cyrene pretreatment preserves the chemical structure of CAMs, preventing lithium loss and enabling the potential for direct recycling of the treated material.

## Supplementary Material



## Notes

Details include the description and characterization (elemental composition, particle size distribution, and SEM micrographs) of the black masses used in this work and the grades and recoveries of graphite and CAMs after flotation (PDF)
