# Supplementary material for: Evaluation of a Bio-Based Solvent Pretreatment for Sustainable Froth Flotation of Black Mass from Spent Lithium-Ion Batteries
Source: ACS Sustain Resour Manag. 2025 Jun 5;2(6):1021–9. doi: 10.1021/acssusresmgt.5c00058 (PMC12207666; doi:10.1021/acssusresmgt.5c00058)
Supplement: Supplementary file 1 [file rm5c00058_si_001.pdf]

Evaluation of a bio-based solvent pre-treatment for sustainable froth flotation of black mass from spent lithium-ion batteries

Aliza Marie Salces [†, ‡,*], Marc Simon Henderson[§,‖], Alvaro José Rodríguez-Medina[†], Kai Bachmann [#], Elsayed Oraby[§], Chau Chun Beh[§], Martin Rudolph[†], Jacques Eksteen[§,‖], Anna Vanderbruggen[‡,*]

[†] Helmholtz Zentrum Dresden Rossendorf (HZDR), Helmholtz Institute Freiberg for Resource Technology (HIF), Chemnitzer Straße 40, 09599 Freiberg, Germany

[‡] Université de Lorraine, GeoRessources, 54000 Nancy, France

[§] Western Australian School of Mines: Minerals, Energy and Chemical Engineering, Curtin University, Perth, Western Australia 6102, Australia

[‖] Future Battery Industries CRC, Bentley, Western Australia 6102, Australia

[#] ERZLABOR Advanced Solutions GmbH, Chemnitzer Straße 40, 09599 Freiberg, Germany

[*] Corresponding author. Email address: a.salces@hzdr.de, anna.vanderbruggen@univ-lorraine.fr

Supporting Information

Table S.1 Pristine powders and black masses used for investigation.

Type	Description	Supplier/Preparation
Pristine material	NMC	$LiN_{i0.33}Mn_{0.33}Co_{0.33}O_2$ (NMC-111, MSE supplies, Product No. PO0126)
	Anode Graphite	Spheroidized natural graphite (ProGraphite GmbH, product No. 1112-1)
Single chemistry black mass (M-BM)	Single chemistry black mass containing a 80:20 mass ratio of separately crushed cathode and anode active materials<br>Anodic material: Graphite with water soluble SBR-CMC binder + Cu foils<br>Cathodic material: NMC with PVDF binder + Al foils	Prismatic hard case LIB cell type

Mixed chemistry black mass (M-IBM)	Mixed chemistry black mass originating from batteries in consumer electronics<br>Anodic material: graphite with PVDF binder + Cu foils<br>Cathodic material: mix of NMC, LCO, (min. LFP) with PVDF binder and Al foils	Undisclosed mechanical processing by Envirostream Australia Pty Ltd.

Table S.2 Complete elemental compositions of BM and IBM. Al, Co, Cu, Li, Mn, Ni from ICP-OES, and C from total combustion. NMC is reported as the sum of Ni+Mn+Co.

	**C, %**	**Al, %**	**Co, %**	**Cu, %**	**Li, %**	**Mn, %**	**Ni, %**	**Fe, %**	**Others (O, F, P)**	**NMC, %**
**M-BM**	18.7	0.9	15.7	1.6	5.6	15.1	15.3	-	27.1	46.1
**MC-BM**	18.2	0.9	16.7	1.6	5.3	15.1	16.3	-	25.9	48.1
**MT-BM**	19.5	1.2	17.0	1.8	5.7	15.4	16.7	-	22.7	49.1
**M-IBM**	39.3	3.4	12.9	2.1	2.8	3.3	10.4	0.1	25.8	26.6
**MC-IBM**	39.9	3.4	12.4	1.9	2.4	3.3	10.2	0.1	26.5	25.9
**MT-IBM**	44.4	4.0	15.4	2.4	3.2	4.0	12.1	0.4	14.5	31.5

Table S.3 Particle size distribution obtained by laser diffraction of the pristine particles and black masses.

Samples	$d_{10,3}$ µm	$d_{50,3}$ µm	$d_{90,3}$ µm
NMC (pristine)	3.9 ± 0.2	10.7 ± 0.6	15.9 ± 0.7
Graphite (pristine)	4.7 ± 0.4	10.2 ± 0.7	15.2 ± 0.9
Single chemistry BM	5.8 ± 0.1	20.8 ± 2.0	79.3 ± 3.7
Mixed chemistry IBM	7.4 ± 0.5	24.1 ± 1.1	210 ± 17.1

Table S.4 Grade and recovery of graphite and Ni+Co+Mn (CAMs) in the O/F product.

Black mass	With attrition - Graphite		With attrition – CAMs	
	Grade, %	Recovery, %	Grade, %	Recovery, %
M-BM	24.3	89.6	44.4	63.0
MC-BM	32.3	91.0	40.2	42.6
MT-BM	70.4	91.4	15.4	7.1
M-IBM	50.7	93.3	21.5	58.3
MC-IBM	56.2	88.3	19.1	41.0
MC-IBM Reuse	60.0	83.1	17.4	40.0
MT-IBM	73.7	97.9	11.6	21.1

Table S.5 Grade and recovery of graphite and Ni+Co+Mn (CAMs) in the U/F product.

Black mass	With attrition - Graphite		With attrition – CAMs	
	Grade, %	Recovery, %	Grade, %	Recovery, %
M-BM	6.07	10.4	56.2	37.0
MC-BM	3.53	9.0	59.8	57.4
MT-BM	2.20	8.6	66.1	92.9
M-IBM	10.04	6.7	42.2	41.7
MC-IBM	11.42	11.7	42.0	59.0
MC-IBM Reuse	15.81	16.9	36.6	60.0
MT-IBM	2.28	2.1	62.2	78.9

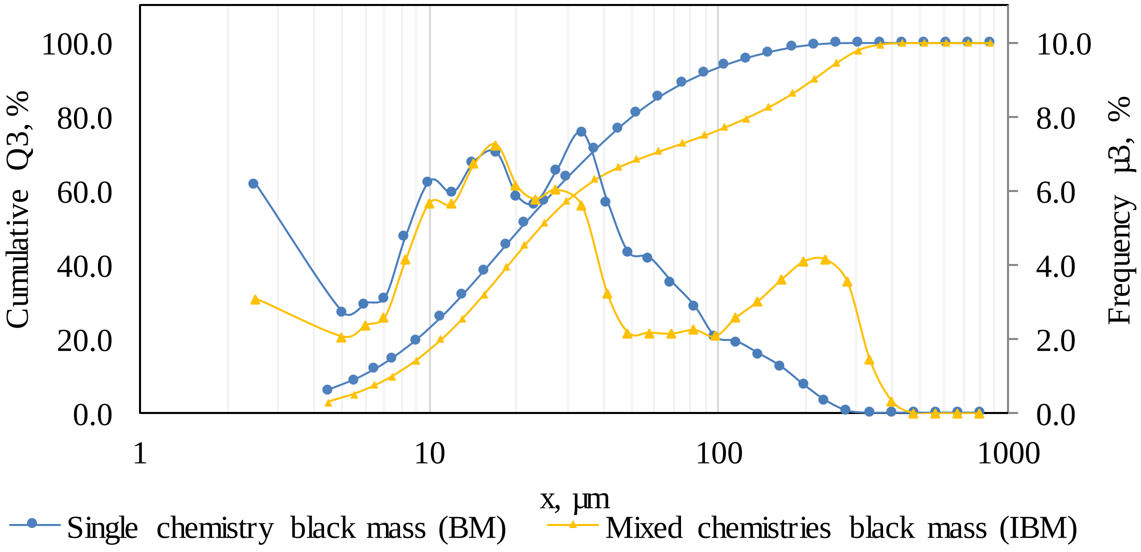

Figure S.1 Particle size distribution. Size range of measurement: 4.5 µm - 875µm.

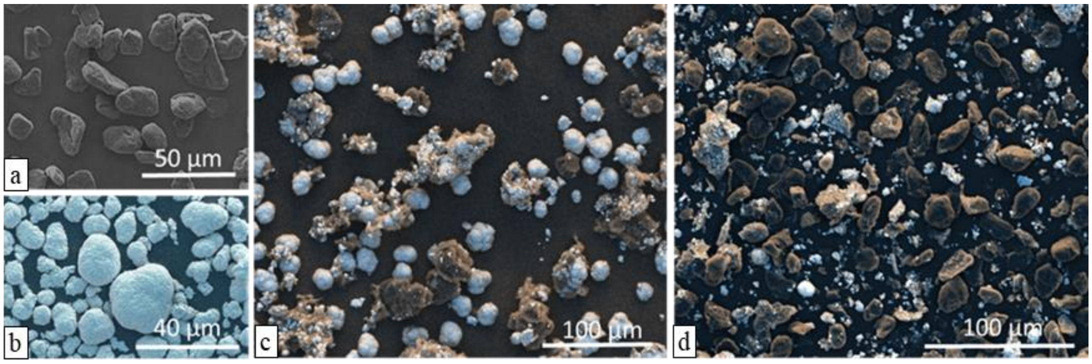

Figure S.2 False-colour SEM images: (a) pristine graphite, (b) pristine NMC, (c) BM and (d) IBM with graphite (black) and CAMs (blue).

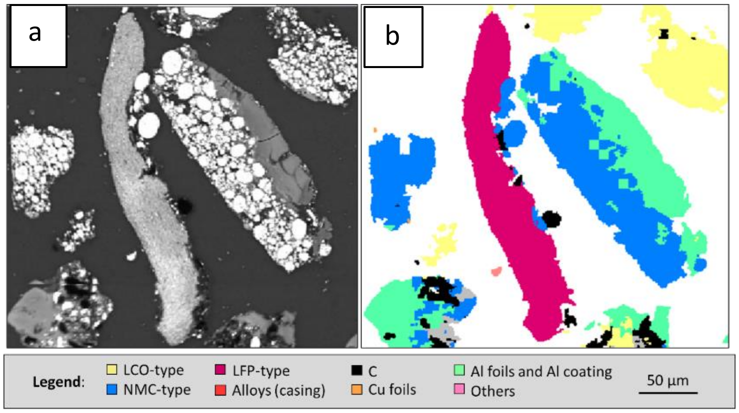

Figure S.3 (a) BSE image and (b) processed image by MLA for the mixed chemistries black mass showing the presence of LFP particle.

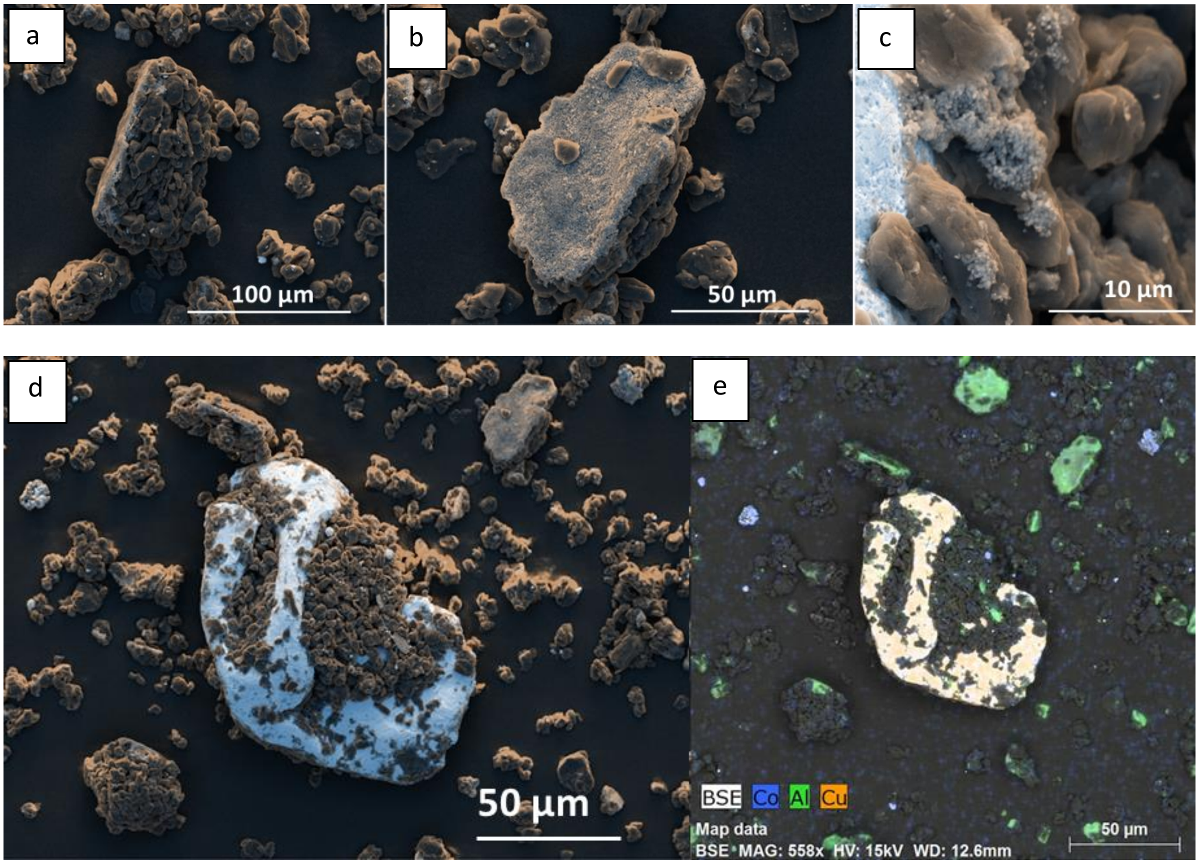

Figure S.4 False-colour SEM images on lose powder of single chemistry black mass (BM) showing (a-d) Al coating made of ultrafine agglomerated particles. (e)EDX mapping confirms that this component is made of Al.

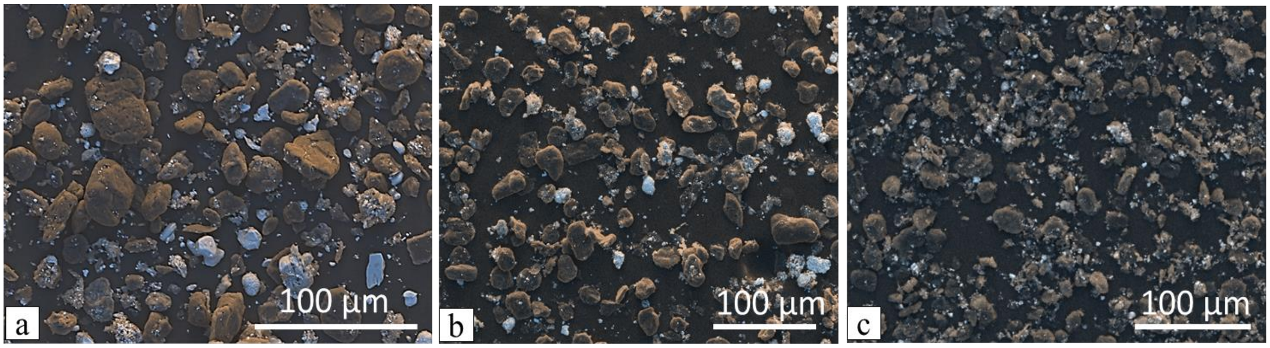

Figure S.5 False-colour SEM images of the rougher's first concentrate in: (a) M-IBM, (b) MC-IBM and (c) MT-IBM. All with attrition pre-treatment.
